# CNTs@ZIF-67 conductive composite nanoparticles enhance the electromagnetic shielding performance of tough organic hydrogels

**DOI:** 10.1016/j.isci.2026.114635

**Published:** 2026-01-07

**Authors:** Zhi Lei, Daohai Zhang, Yupeng Hu, Teng Zhou, Kunlan Diao, Dongju Liu, Anmin Song, Kaixiang Zou, Shuhao Qin

**Affiliations:** 1 School of Chemical Engineering of Guizhou Minzu University, Guizhou, Guiyang 550025, China; 2 National Engineering Research Center for Compounding and Modification of Polymer Materials, Guizhou, Guiyang 550014, China

**Keywords:** electromagnetic shielding, CNTs, ZIF-67, organic hydrogel

## Abstract

With the widespread use of electronic devices, electromagnetic radiation pollution has become increasingly severe, posing potential threats to human health and the ecological environment. This study successfully prepared a tough, adhesive, green, and multifunctional semi-interpenetrating network organic hydrogel using a three-step method, and enhanced its electromagnetic shielding performance by incorporating CNTs@ZIF-67 composite nanoparticles. An environmentally friendly semi-interpenetrating network organic hydrogel was constructed using polyvinyl alcohol (PVA) and bio-based gelatin (Gel). Through an *in situ* growth method, ZIF-67 was grown on the surface of carbon nanotubes (CNTs) to prepare CNTs@ZIF-67 composite nanoparticles. Using a one-pot approach, these composite nanoparticles were dispersed in a precursor solution containing gelatin and PVA, followed by cyclic freeze-thaw processing to fabricate a novel hydrogel material that combines high toughness with outstanding electromagnetic shielding performance. When incorporating 50 mg of CNTs@ZIF-67, the organic hydrogel achieves a conductivity of 0.512 ± 0.026 S/m, a compressive modulus of 41.8 ± 2.09 kPa, a compressive deformation rate of 66.33% ± 3.01%, a moisture content of 88.99% ± 0.89%, and a water absorption rate of 265.92% ± 1.33%. The PGZC1 organic hydrogel with a thickness of 3 mm exhibits an average electromagnetic interference shielding effectiveness (EMI SE) of 33.32 ± 1.67 dB within the X-band (8.2–12.4 GHz), thus providing an innovative material solution for mitigating electromagnetic wave pollution.

## Highlights

• The 3D porous network offers multiple paths to reflect and attenuate electromagnetic waves
• ZIF-67’s magnetic loss and CNTs’ dielectric loss boost EMI shielding performance
• Low-cost semi-interpenetrating Gel/PVA hydrogel formed using green, affordable materials
• This work delivers an adaptable, wearable, flexible material for EM-protected electronics

## Graphical abstract

**Figure:**
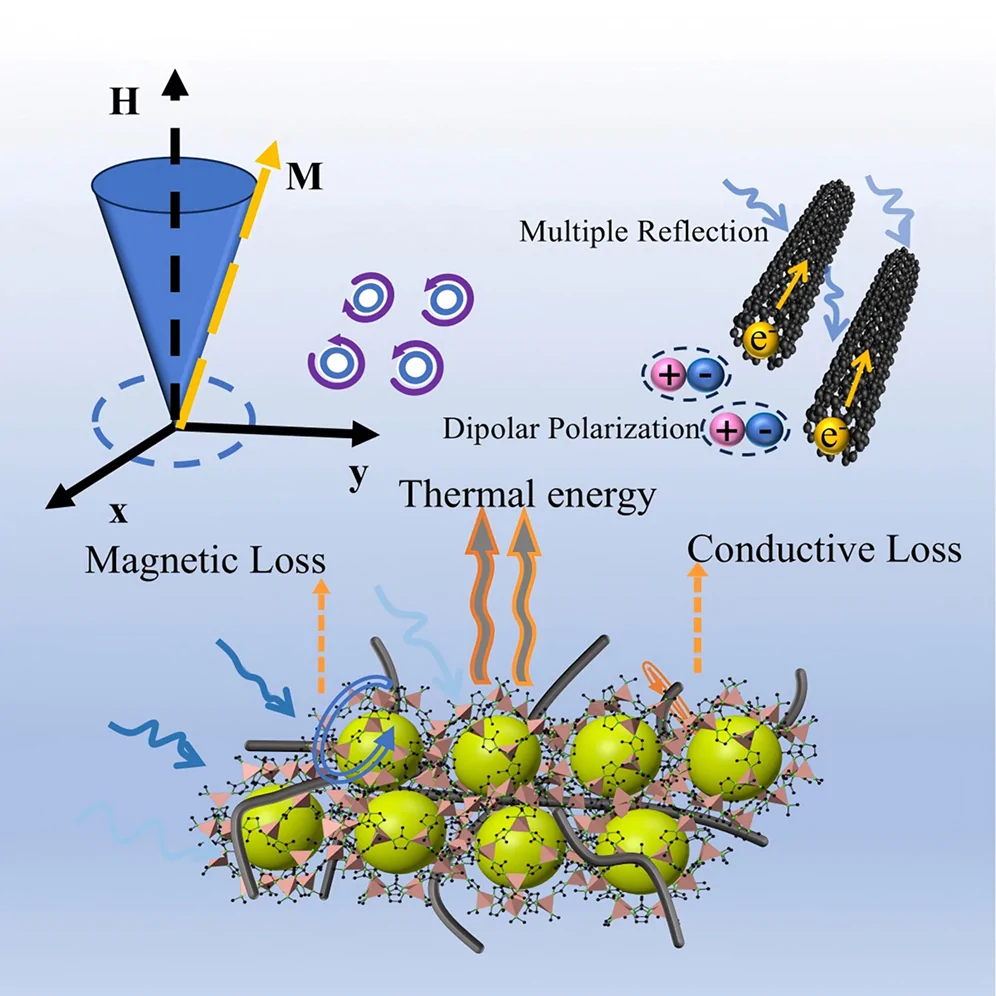

## Teaser

Physics; Electromagnetic field; Applied sciences

## Introduction

In the wake of the swift progress of modern technology, electronic devices have proliferated and become an integral part of people’s daily existence. However, during the use of electronic devices, electromagnetic interference (EMI) is inevitable. This will not only interfere with other electronic devices in operation but could potentially endanger human well-being.^1^^,^^2^^,^^3^^,^^4^ For example, in the medical field, the measurement accuracy of sophisticated medical equipment can be reduced due to interference from electromagnetic waves, thus affecting the diagnosis and treatment of patients^5^^,^^6^; In the field of aerospace, interference from electromagnetic waves can pose a threat to the flight safety of aircraft.^7^ Therefore, developing a highly efficient electromagnetic shielding material is of great importance.

Carbon-based materials, such as graphene, carbon nanotubes (CNT), and carbon nanofibers (CNF),^8^ have advantages such as being lightweight and having good electrical conductivity.^9^^,^^10^ Luo et al.^11^ prepared a Janus cellulose composite film with a multi-layered structure through layer-by-layer vacuum filtration. When there is a CNT/CNF layer containing 50 mg of CNF, the maximum electromagnetic shielding performance of the composite film can reach 20.5 dB. Metal-organic frameworks (MOFs) represent a category of versatile materials characterized by their well-organized porous architectures, which form through the self-assembly of metal ions (or clusters) and organic ligands.^12^^,^^13^ In recent years, because they possess unique physical and chemical properties, MOFs and their carbon-based derivatives have shown significant promise in applications related to electromagnetic shielding and absorption.^14^^,^^15^ Wu et al.^16^ carried out liquid-phase co-deposition. Through this process, copper (Cu) and iron (Fe) bimetallic heterostructure nanoparticles were formed on the surface of CNTs and prepared a Cu/Fe@CNT/SA/PDMS composite material with a three-layer structure. When the content of Cu/Fe@ CNT is 0.1 wt%, its electromagnetic shielding performance reaches 21 dB. Jin et al.^17^ prepared NiCo@NPC@CF - 800 fibrous nanocomposites composed of micro-nano-hierarchical porous carbon structures along with flower-like NiCo@C particles. Ni/Co-MOF particles and biomass cotton serve as precursors for its formation. The overall shielding performance (SET) of this substance achieved 30.58 dB. To endow conventional electromagnetic shielding materials with adaptive properties, we incorporated them into a hydrogel matrix system. Hydrogel is a kind of flexible material with a high water content and good flexibility. Its excellent biocompatibility and ease of processing and modification make it popular among functional materials researchers.^18^^,^^19^ By introducing conductive materials, such as metal nanoparticles, CNTs, graphene, and other materials into the spatial structure of the hydrogel, a hydrogel with electromagnetic shielding performance can be prepared.^20^ Lu et al.^21^ synthesized polypyrrole nanotube-incorporated hydrogels, achieving an EMI shielding effectiveness (SE) of 21 dB with a 2 mm-thick hydrogel sample. This hydrogel not only retains its inherent flexibility and extensibility but also demonstrates excellent electromagnetic shielding performance, thereby meeting the stringent requirements of modern electronic devices for EMI shielding materials.

Despite the progress made by previous researchers in developing composites with favorable electromagnetic shielding performance, these materials still suffer from notable limitations, including complex preparation processes, high raw material costs, inadequate flexibility, and restricted applicability. To address these limitations, it is imperative to develop green, tough, and adhesive gel materials utilizing low-cost biomass components for achieving novel materials with superior electromagnetic shielding performance. This study developed a semi-interpenetrating network composite gel system by employing environmentally friendly polyvinyl alcohol (PVA) and bio-gelatin (Gel), utilizing hydrogen bonding for network construction. The abundant oxygen-containing groups in both raw materials enable adhesion to skin or other substrates through hydrogen bonding and intermolecular forces, thereby achieving wearable functionality. Furthermore, the incorporation of highly conductive CNTs@ZIF-67 composite nanoparticles into the gel system significantly enhances its electrical conductivity. The CNTs@ZIF-67 hybrid exhibits dual electromagnetic wave absorption mechanisms through both magnetic loss and dielectric loss, effectively dissipating the electromagnetic energy as heat. Simultaneously, the organic hydrogel’s network structure provides anchoring sites for the composite nanoparticles, while its three-dimensional porous architecture enables multiple reflections and attenuation of electromagnetic waves, thereby significantly enhancing the overall electromagnetic shielding capability of the material.^22^ In this study, a green, adhesive, moldable, organic hydrogel with high toughness and strong electromagnetic shielding properties was successfully prepared by *in situ* growth method, one-pot method, and cyclic freezing method. These findings highlight the organic hydrogel’s significant potential for broad applications in smart wearable technologies and electromagnetic shielding, revealing important development opportunities.

## Results and discussion

### Morphology and characterization of the hydrogel

Scanning electron microscopy (SEM) was employed to examine the surface morphology of the samples. As evidenced by the SEM image of as-synthesized ZIF-67 (Figure 2A), the obtained ZIF-67 crystals exhibit well-defined rhombic dodecahedron morphology.^23^ As observed in Figure 2B, ZIF-67 grows on the CNTs through *in situ* deposition, confirming the successful preparation of the CNTs@ZIF-67 composite material. The PVA/Gelatin (PG) hydrogel forms a three-dimensional network structure (Figure 1C), resulting from polymer chain entanglement during gelation. Concurrently, hydrogen bonding between functional groups (e.g., amino and hydroxyl groups) on the polymer chains induces molecular crosslinking, ultimately creating the porous architecture.^24^ These three-dimensional networks and hydrophilic functional groups (e.g., –OH, –COOH) collectively contribute to the hydrogel’s exceptional water retention and absorption capacities.^25^ The incorporation of ZIF-67, CNTs, and CNTs@ZIF-67 into the PG hydrogel matrix significantly reduces the porosity of the hydrogel’s internal architecture. However, upon incorporation of ZIF-67 (Figure 2D), the hydrogel exhibits a smoother surface morphology with complete disruption of its internal three-dimensional network structure. The Co^2+^ ions exhibit electrostatic attraction with electron-rich groups on the polymer chains, inducing chain segment approximation and subsequent aggregation.^26^ Due to their nanoscale dimensions and high specific surface area, CNTs effectively infiltrate the hydrogel’s network architecture.^27^ Due to their nanoscale dimensions and high specific surface area, CNTs effectively infiltrate the hydrogel’s network architecture, resulting in significant pore size reduction in PG hydrogel (Figure 2E). However, in Figure 2F, the pore structure of the PGCZ1 hydrogel exhibits both a higher quantity and significantly larger dimensions compared to the PVA/Gelatin/CNTs (PGC) and PVA/Gelatin/ZIF-67 (PGZ) groups. This is because the oxygen anions on carbon nanotube surfaces neutralize the electrostatic effects of Co^2+^ ions, creating an electrostatic confinement effect on ZIF-67. This phenomenon reduces the influence on corresponding charges of polymer molecular chains, thereby weakening the electrostatic interactions between the CNTs@ZIF-67 composite and the hydrogel matrix.^28^^,^^29^

**Figure 1:**
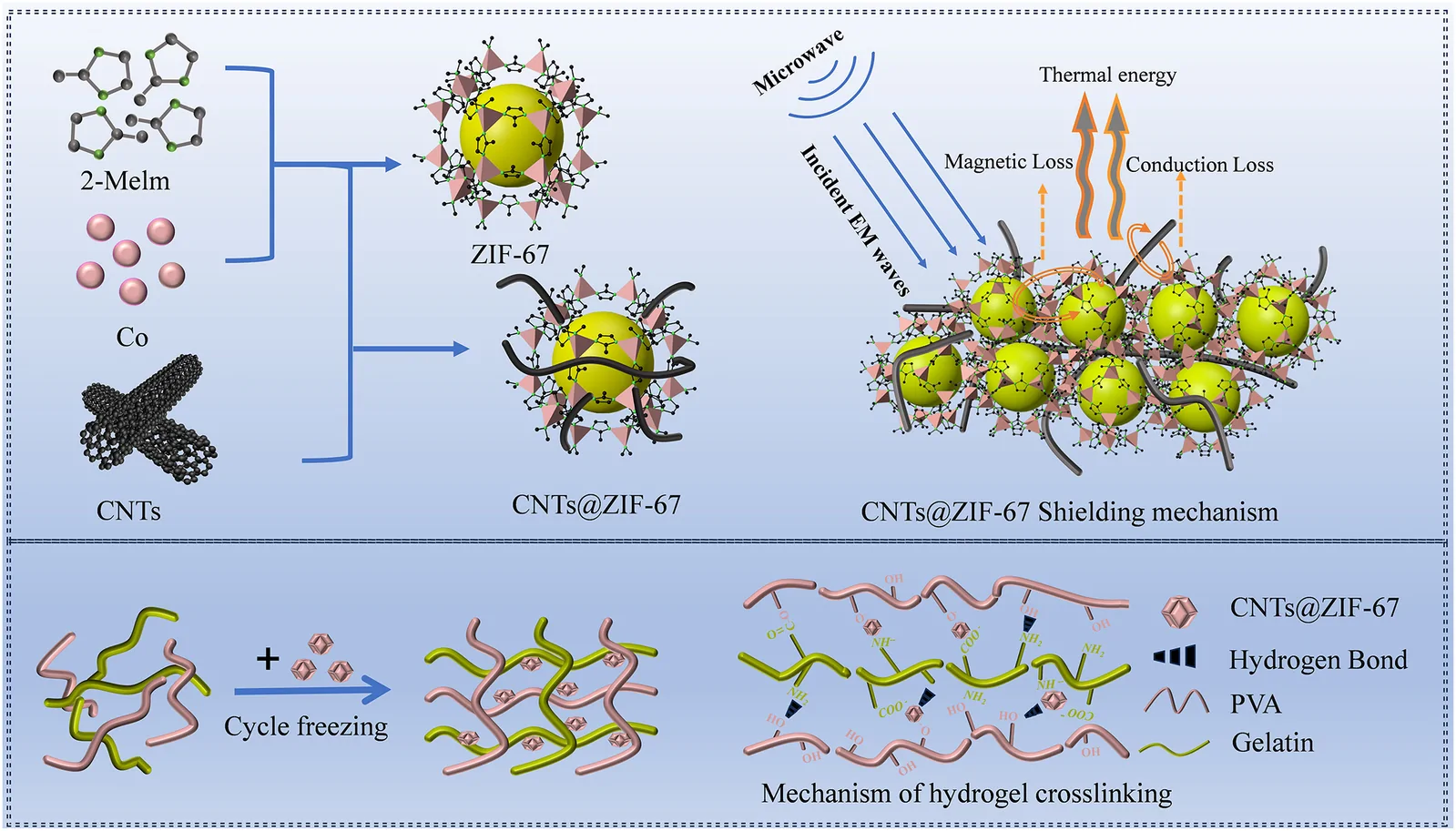
Illustration of the fabrication procedure

**Figure 2:**
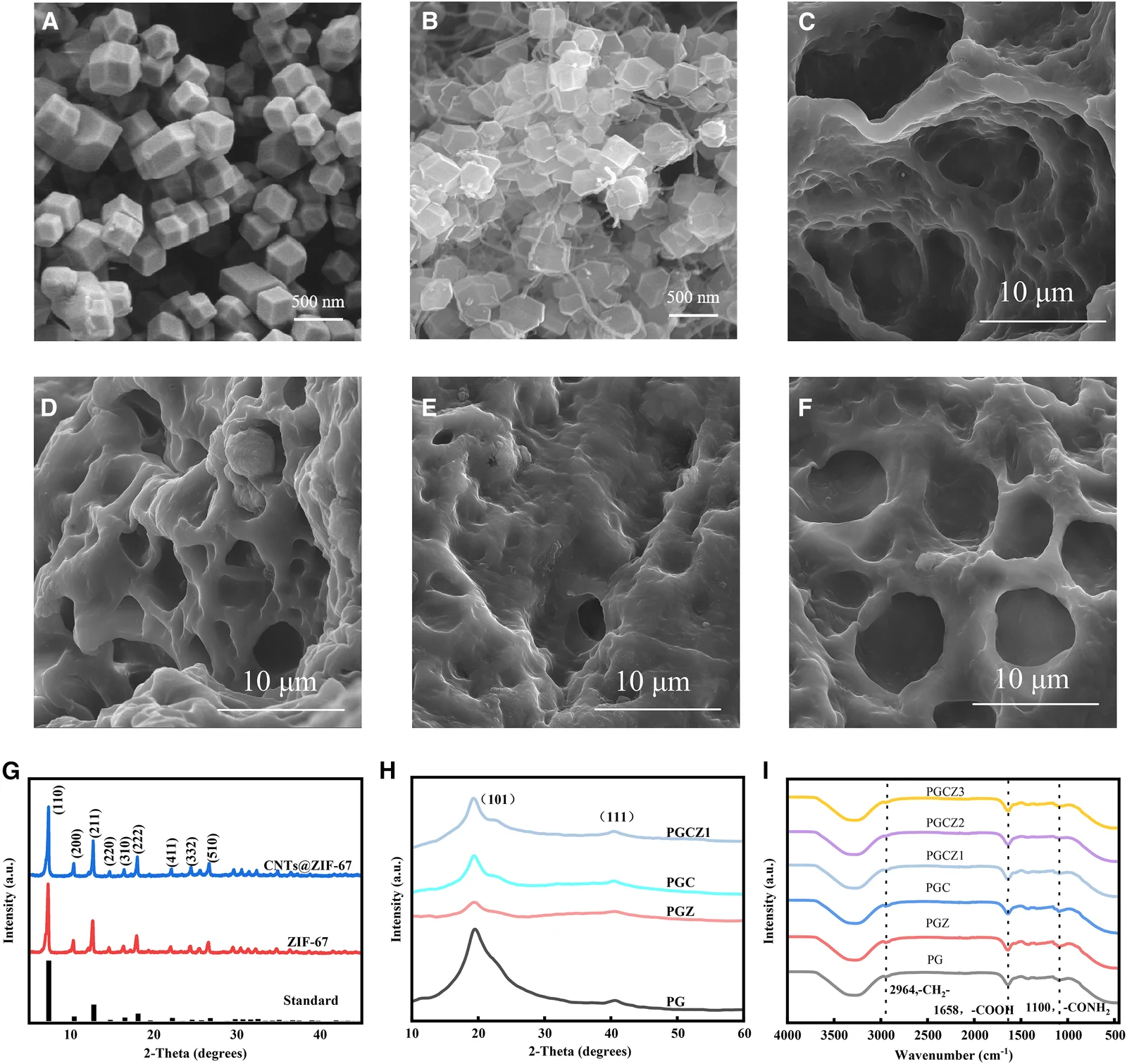
Microscopic structural characterization of CNTs@ZIF-67 and hydrogels (A and B) SEM images of (A) ZIF-6 and (B) CNTs@ZIF-67. (C–F) SEM images of PG hydrogel, PGZ hydrogel, PGC hydrogel, and PGZC1 hydrogel, respectively. (G) XRD patterns of as-synthesized ZIF-67, CNTs@ZIF-67, and standard ZIF-67 reference. (H and I) (H) XRD patterns and (I) FTIR spectra of the hydrogels.

The X-ray diffraction (XRD) pattern of the as-prepared sample shows perfect consistency with the standard reference pattern of ZIF-67 (Figure 2G). The characteristic peaks at 2θ = 7.28°, 10.34°, 12.87°, 14.56°, 16.35°, 17.93°, 22.1°, 24.42°, 26.58°, and 29.5° map onto the (011), (002), (112), (022), (013), (222), (114), (233), (134), and (044) crystal planes of ZIF-67. As demonstrated in figure, the characteristic peaks of the sample are sharp and distinct, indicating that the lattice structure of the prepared ZIF-67 powder is relatively complete.^30^ For the hydrogel, the diffraction angle 2θ = 19.41° map onto the (101) crystal plane and 2θ = 40.73° map onto the (111) crystal plane (Figure 2H). The magnitudes of the diffraction angles at these two crystal planes have not changed, but the widths of the diffraction peaks have narrowed, underscoring that the size of the crystal grains has been altered due to the filling.^31^ The cobalt ions in ZIF-67 have the greatest impact on the changes of these two crystal planes.

From the Fourier transform infrared (FTIR) spectral image (Figure 2I), it can be seen that the introduction of CNTs and ZIF-67 did not introduce new functional groups into the hydrogel. Instead, similar to the PVA/Gel hydrogel groups, an absorption peak of methylene (–CH_2_–) appears at 2,964 cm^−1^; an absorption peak of carboxyl (–COOH) appears at 1,658 cm^−1^; and an absorption peak of amide (–CONH_2_) appears at 1,100 cm^−1^. This indicates that within the hydrogel system, CNTs, ZIF-67, and CNTs@ZIF-67 do not form new chemical bonds with the PG matrix, but rather physically disperse within the PG derived precursor framework.

### Structural characteristics of the hydrogel

To further characterize the sample, X-ray photoelectron spectroscopy (XPS) analysis was conducted to identify the sample’s component element, oxidation states, and atomic ratios.^28^ As can be seen from (Figures 3D–3G), the C1s peaks of CNTs@ZIF-67 at 284.4 eV and 285.2 eV correspond to C–C bonds and C–N bonds,^32^ respectively; O1s spectra showed the presence of oxygen vacancies (O Vac, 531.8 eV),^33^ The N 1s energy spectrum showed that the characteristic peak of imidazole-N in ZIF-67 was 398.9 eV, and the characteristic peak of C=N bond in 2-methylimidazole was 400.1 eV.^34^ The Co 2p spectrum had two main and two satellite peaks, which were Co 2p3/2 (781.7 eV) and Co 2p1/2 (797.1 eV), and Co 2p3/2 sat (785.7 eV) and Co 2p1/2 sat (803.2 eV),^35^ which further demonstrated the successful preparation of CNTs@ZIF-67. Elemental characterization of ZIF-67, PGZ, and PGZC1 confirmed identical elemental composition across all three components (Figure 3A). For PGZ and PGZC1, the characteristic peaks of cobalt were not significantly detected in the XPS spectra due to their relatively low Co content. The incorporation of CNTs@ZIF-67 into the PG matrix induces electronic interactions between the composite’s metal ions and characteristic functional groups of PG, resulting in electron density redistribution and consequent binding energy shifts (Figures 3B and 3C). In Figures 3H and 3I, the Co2p3/2 energy levels in the hydrogels were 781.7 eV and 781.5 eV, respectively, with an increase of 0.3 eV and 0.1 eV, respectively. The Co 2p1/2 energy levels were 797.8 eV and 797.5 eV, respectively, with an increase of 0.7 eV and 0.4 eV, respectively. This indicates that groups such as hydroxyl (–OH) and carboxyl (–COOH) groups in the hydrogel may undergo coordination with Co^2+^, increasing the electron density around the cobalt ions, and thus increasing the binding energy.^36^

**Figure 3:**
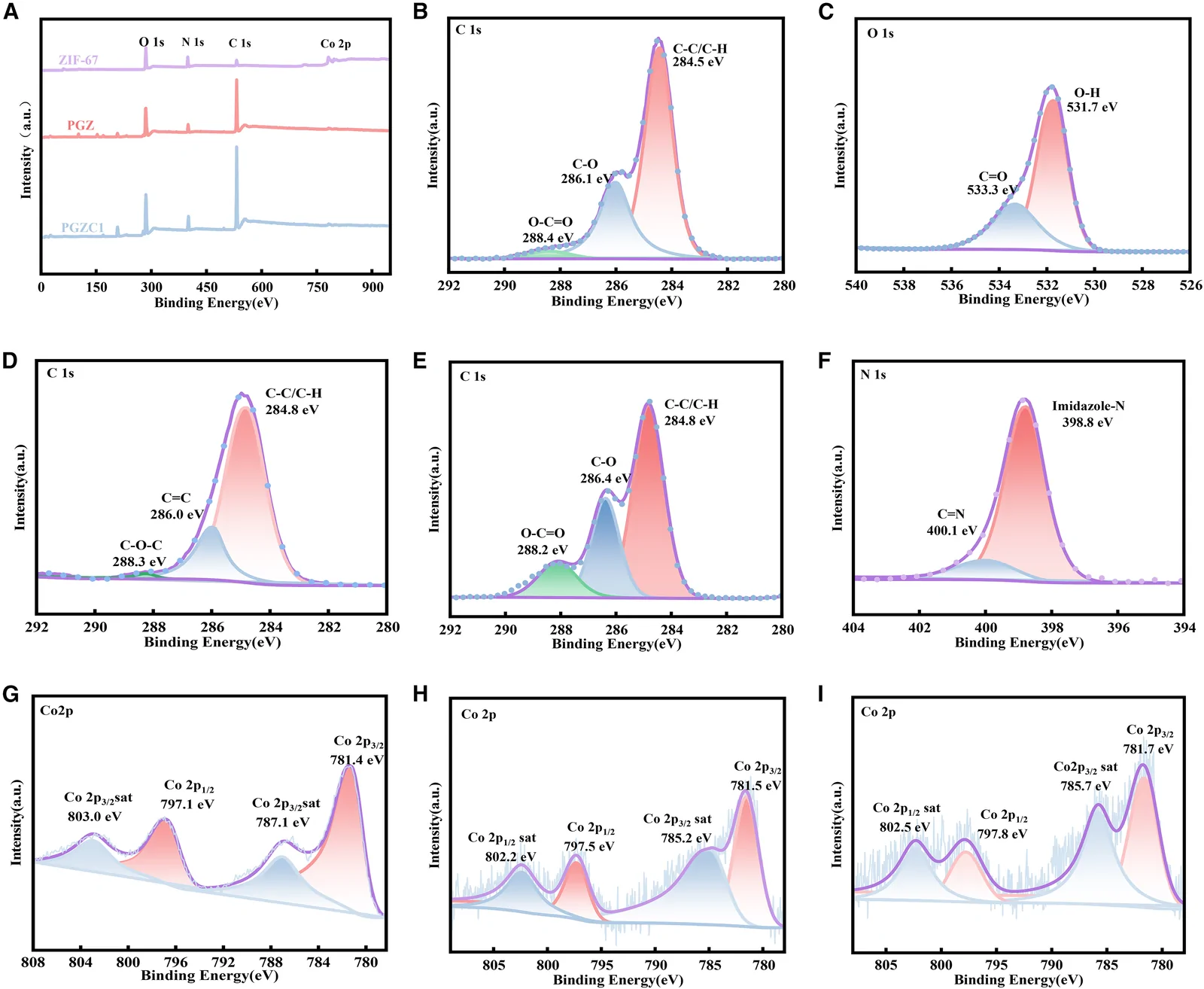
XPS characterization of CNTs@ZIF-67 and hydrogels (A) Survey X-ray photoelectron spectroscopy (XPS) spectra of ZIF-67, PGZ, and PGZC1. (B and C) High-resolution C 1s spectra of PG and PGZC1. (D–F) The C1s, N1s, and O1s spectra of ZIF-67. (G–I) Co 2p spectra for ZIF-67, PGZ, and PGZC1.

### Performance tests of the hydrogel

Differential scanning calorimetry (DSC) was employed to investigate the crystallization and melting temperatures of the hydrogels. As shown in Figure 4A, the incorporation of CNTs, ZIF-67, or CNTs@ZIF-67 did not alter the crystallization temperature of PG. This thermal stability can be attributed to the low additive concentrations, which were insufficient to significantly perturb the crystallization behavior of PG.^37^ In the melting temperature curve, we observe that the melting temperature range exhibits significant stability, with the melting process occurring approximately between −3.5°C and 12.0°C (Figure 4B). This is because the polymer can only partially crystallize, consisting of both crystalline and amorphous regions.^38^

**Figure 4:**
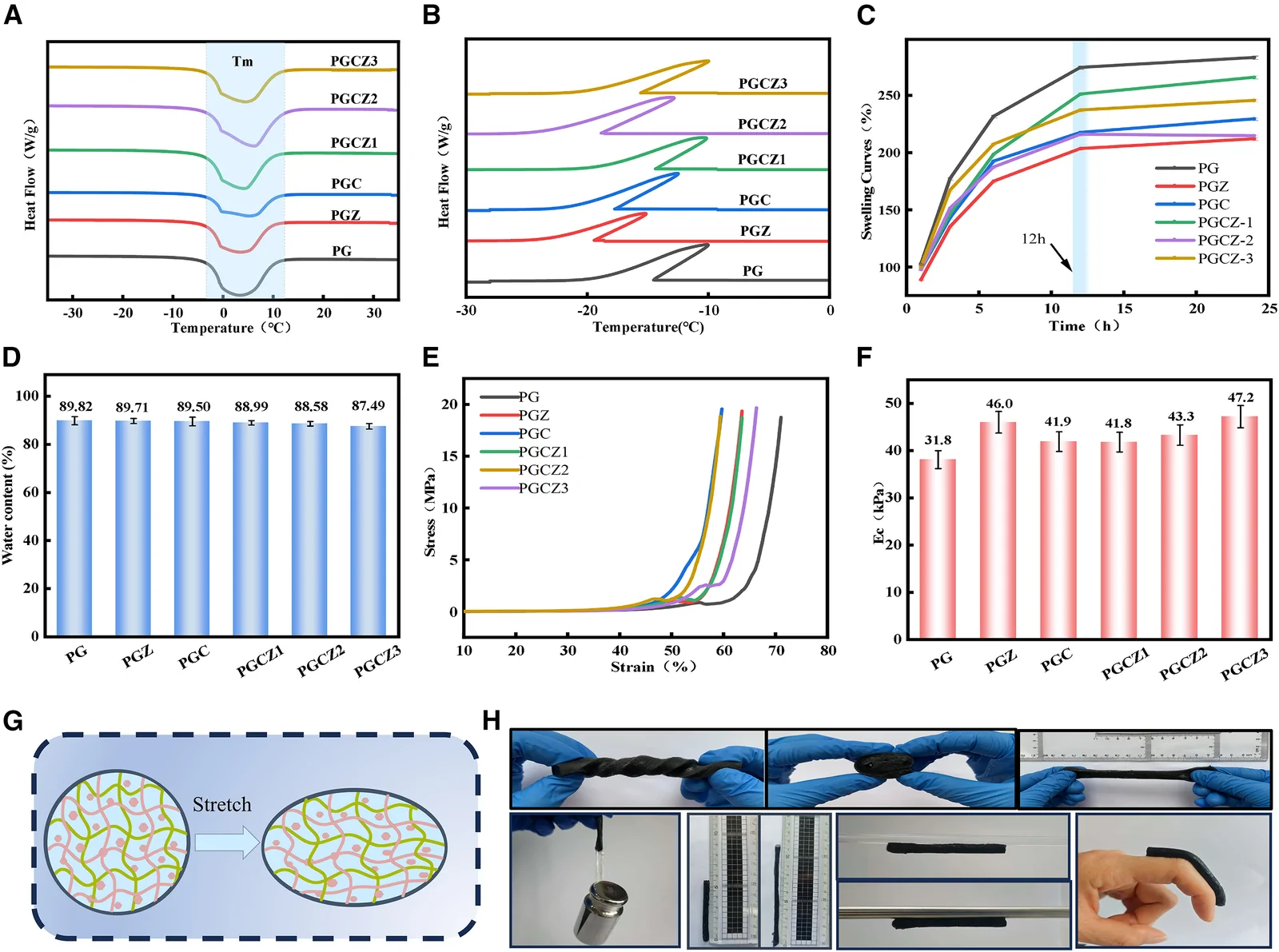
Physical properties of hydrogels (A and B) (A) Dissolution temperature and (B) crystallization temperature profiles of the hydrogels. (C and D) (C) Water content and (D) water absorption kinetics of the hydrogels. (E and F) (E) Compressive stress-strain curves and (F) compressive modulus of the hydrogels. (G) Schematic illustration of hydrogel stretching. (H) PGCZ1 hydrogel toughness and adhesion.

In the water content test (Figure 4C) and water solubility test (Figure 4D), it can be observed that the hydrogel maintained a high swelling ratio within the first 12 h. Furthermore, doping did not significantly affect its water content, which decreased slightly from 89.82% to 88.99% ± 0.89%. In contrast, a more noticeable change occurred in water absorption performance, with the absorption rate decreasing from 283.23% to 265.92% ± 1.33%. Notably, these variations did not compromise the doped hydrogel’s characteristic high water absorption capacity and high water content.

In the compression performance tests of the materials, according to the compressive stress-strain curves (Figure 4E), the PG exhibited a compressive strength of 18.75 MPa and a compressive strain of 71.07%. For the electromagnetic shielding hydrogel incorporating CNTs@ZIF-67, the compressive strength reached 19.67 MPa with a compressive strain of 66.33% ± 3.01%. Although the compressive strain of the hydrogel decreased after doping, the compressive modulus exhibited an opposite trend (Figure 4F). The PG showed the lowest compressive modulus at 38.1 kPa. This can be attributed to the incorporation of CNTs and ZIF-67, which reduces the compressive strain but enhances the compressive modulus of the hydrogel.^37^ This behavior can be explained by the uniform dispersion of dopants within the hydrogel matrix and their interfacial interactions with the intrinsic polymer chains, which collectively enhance the polymer’s crosslinking density. These interactions effectively constrain the mobility of polymer chains, limiting their extensibility.^38^ As a result, the doped hydrogel demonstrates decreased compressive strain but improved compressive modulus. As observed during the stretching process (Figure 4G), the polymer chains align along the loading direction while the perpendicular segments aggregate toward the center. Through simple tensile, bending, and twisting tests (Figure 4H), the PGZC1 electromagnetic shielding hydrogel demonstrates remarkable deformability without structural failure under mechanical stress. PGCZ1 organic hydrogel with a 0.5 cm diameter could lift a 200 g weight without fracture and maintained stable adhesion to paper, iron rods, and glass rods for extended periods without detachment. These results demonstrate that this electromagnetic shielding hydrogel possesses excellent flexibility and adhesive properties, showing promising potential for flexible wearable applications.

### Electromagnetic shielding performance of the hydrogel

Testing the hydrogel’s conductivity via a four-probe resistance tester (Figure 5F), we can observe that the introduction of CNTs and ZIF-67 improves the electrical conductivity of the hydrogel. Moreover, as the proportion of CNTs increases, the hydrogel’s electrical conductivity is also enhanced.^39^^,^^40^ Owing to the high conductivity of CNTs, the electrical conductivity of the hydrogel increases from 0.34 S/m (PG) to 0.62 S/m, representing an 82.35% enhancement relative to the pristine PG.

**Figure 5:**
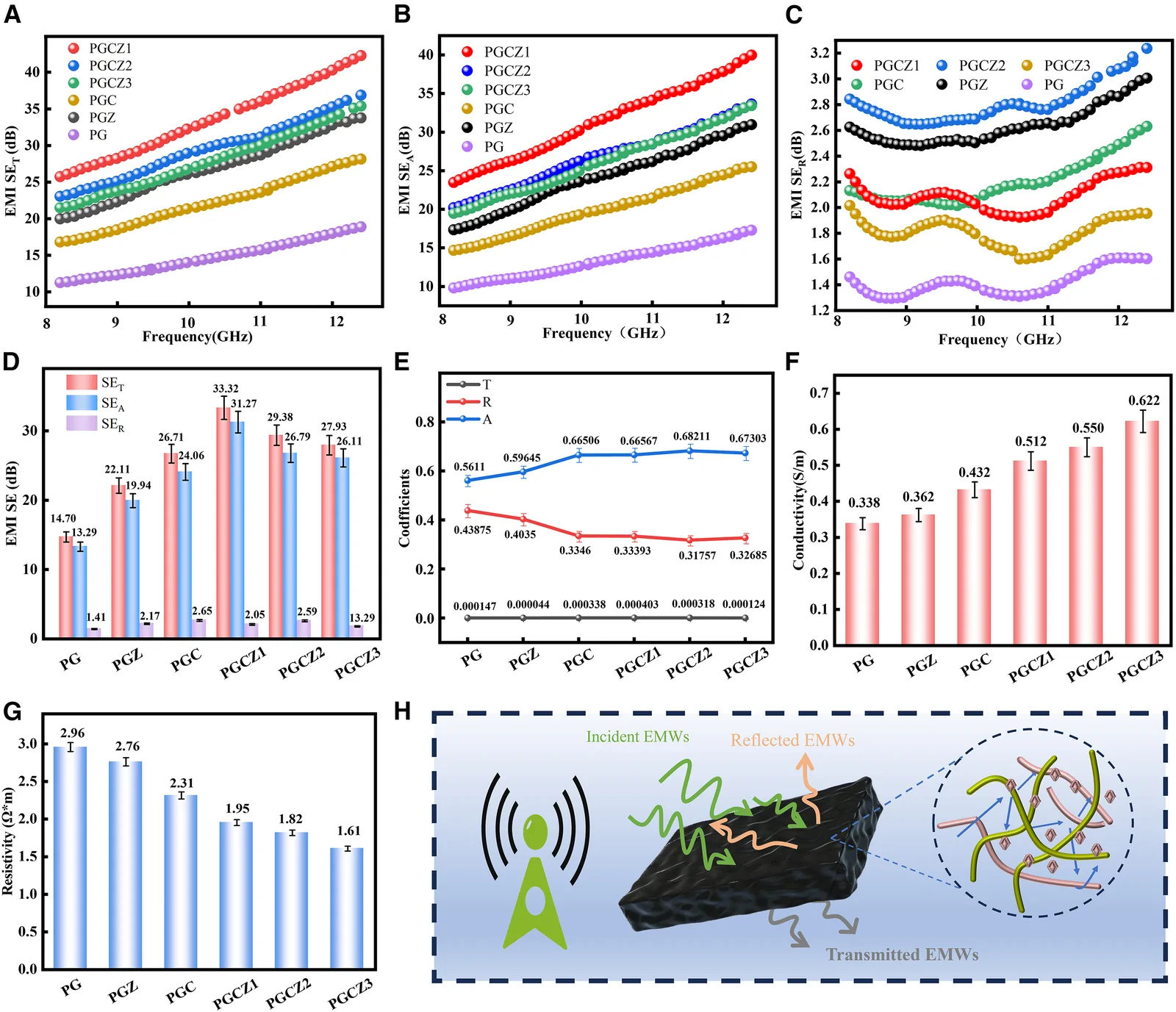
Electromagnetic shielding performance of hydrogels (A–C) Frequency-induced changes in the SE_T_, SE_R_, and SE_A_ of the hydrogel. (D) The average SE_T_, SE_R_, and SE_A_ of the hydrogel. (E) The T-R-A coefficients of the hydrogel. (F and G) Hydrogel’s capacity to conduct electricity. (H) The hydrogel’s mechanism for electromagnetic field shielding.

Generally speaking, the EMI shielding performance is affected by reflection, absorption, and multiple reflections, which correspond to mobile charge carriers, electric dipoles, and internal interfaces/surfaces.^41^^,^^42^ In addition, the porous framework both lowers the density of the material and offers an extensive surface area and accessible active sites. These active sites facilitate the scattering or repeated reflections of electromagnetic waves (EMWs), leading to energy dissipation.^43^ Because CNTs and ZIF-67 possess electromagnetic shielding properties, their synergistic effect can effectively improve the magnetic loss and dielectric loss of the hydrogel.^44^^,^^45^ It can be seen from Figures 5A–5C that when CNTs@ ZIF-67 are added, the hydrogel’s electromagnetic SE is notably enhanced, and the SEA (shielding effectiveness of absorption) dominates the electromagnetic shielding performance. This is because the high conductivity of CNTs in hydrogels leads to an increase in the dielectric constant, which consumes the mobile charge carriers, cobalt has a certain absorption capacity for electromagnetic waves.^46^^,^^47^ Within the 8.2–12.4 GHz frequency range, the PG hydrogel achieves an average EMI SE of 14.70 dB. Significantly, the PGZC1 composite outperforms this baseline, with an average SE of 33.32 ± 1.67 dB—marking a 126.7% improvement over the pristine PG. As clearly demonstrated in the PGCZ hydrogel system, the EMI SE progressively decreases with increasing CNT concentration in the CNTs@ZIF-67 filler. This is because CNTs exhibit a high specific surface area and robust van der Waals interactions, which easily lead to agglomeration. As the proportion of CNTs increases, the agglomeration phenomenon intensifies.^48^ With the increase in the proportion of CNTs, the aggregation phenomenon becomes more pronounced. The presence of aggregates disrupts the synergistic effect between CNTs and ZIF-67, thereby weakening the electromagnetic shielding performance.^49^ Meanwhile, excessive aggregation of CNTs within the hydrogel can damage its porous structure, reducing the surface area and active sites of the pores.^44^^,^^50^ In the hydrogel, a positive correlation can be observed between electromagnetic shielding performance and SEA as the frequency of the interfering EMWs increases. Although the reflection efficiency exhibits localized fluctuations—decreasing and increasing in certain ranges—it generally oscillates within a specific bandwidth. This behavior may be influenced by the surface smoothness of the material. All samples exhibited near-zero T values (Figure 5E), indicating their high efficiency in resisting EMW interference.^51^^,^^52^ Meanwhile, the A values were consistently higher than the R values, demonstrating that the primary EMI shielding mechanism was absorption.^53^ With the introduction of materials such as CNTs@ZIF-67 composites, the absorption of electromagnetic waves was enhanced.

The schematic diagram of the electromagnetic shielding mechanism (Figure 5H) further illustrates the EMI shielding mechanism of the hydrogel. PG constructs the three-dimensional structural framework of the hydrogel. When CNTs@ZIF-67 composites are introduced, they become uniformly dispersed within the hydrogel, forming a conductive network that enhances the hydrogel’s electrical conductivity. Meanwhile, the synergistic effect of CNTs@ZIF-67 leads to the formation of relatively independent and complete spherical pore structures within the hydrogel. When electromagnetic waves encounter the material surface, a portion is reflected while the remainder penetrates into the interior. The penetrating waves undergo attenuation as they pass through the conductive walls of the material. Another portion of the reflected waves, upon passing through the material and reaching the next wall layer, gets reflected again and further attenuated.^54^^,^^55^ This structural configuration enhances the probability of multiple reflections and progressive attenuation of electromagnetic waves.

### Conclusion

In this study, a three-step method was employed to successfully fabricate CNTs@ZIF-67 electromagnetic shielding hydrogels by incorporating CNTs@ZIF-67 composites into a three-dimensional network structure composed of PVA and Gel. Based on the experimental performance tests and characterization results, the PGZC1 hydrogel exhibits an average EMI SE of 33.32 ± 1.67 dB (X-band, 8.2–12.4 GHz), an electrical conductivity of 0.512 ± 0.026 S/m, a compressive modulus of 41.8 ± 2.09 kPa, the compression deformation is 66.33% ± 3.01%, the water content is 88.99% ± 0.89%, and the water absorption rate is 265.92% ± 1.33%. The integration of CNTs@ZIF-67 composites not only boosts the hydrogel’s electromagnetic shielding performance and electrical conductivity but also retains its inherent properties, such as water absorption, water retention, ductility, and flexibility. This advanced material demonstrates promising application potential in flexible electronics, wearable devices, and stretchable sensors.

## Resource availability

### Lead contact

Request for further information and/or resources pertaining to this article should be directed to the corresponding author Daohai Zhang: zhangdaohai6235@163.com

### Materials availability

Acrylamide, N,N′-methylene diacrylamide, Ammonium persulfate, N,N,N′,N′-tetramethylethylenediamine, multi-walled carbon nanotubes and poly(3,4-ethyldioxothiophene)-poly(styrenesulfonate) were bought from Aladdin company. Please refer to the key resources table for their details.

### Data and code availability

Data, code, and additional information required to reanalyze the data reported in this study are available from the lead contact upon request.

## Acknowledgments

This work was supported by 10.13039/501100001809National Natural Science Foundation of China Project (52163001), Guizhou Provincial Science and Technology Program Project Grant (Qiankehe Platform Talents-CXTD [2021]005, Qiankehe Platform Talents-GCC [2022]010-1, Qiankehe Platform Talents-GCC[2023]035, Qiankehe Platform Talents-CXTD[2023]003, Qiankehe Platform Talents-KXJZ [2024]022), Guizhou Minzu University Research Platform Grant (GZMUGCZX [2021]01), Central Guided Local Science and Technology Development Funds Project (Qiankehe Zhong Yindi [2023]035), Doctor Startup Fund of Guizhou Minzu University (grant o. GZMUZK [2024] QD77), Guizhou Province Special Fund for innovative capacity building of scientific research institutions (Qiankehe Fuqi [2023]001, Qiankehe Fuqi [2024]002-1),and Guizhou Science and Technology Department Project of Basic Research - ZK [2024] General Project no. 488.

## Author contributions

Z.L., validation, writing – original draft, and writing – review and editing; Y.H., writing – original draft and data curation; T.Z., data curation; K.D., data curation; D.L., data curation; D.Z., A.S., K.Z., and S.Q., funding acquisition and writing – review and editing.

## Declaration of interests

No potential conflict of interest was reported by the authors.

## STAR★Methods

### Key resources table

**Table:** 

REAGENT or RESOURCE	SOURCE	IDENTIFIER
**Chemicals, peptides, and recombinant proteins**		

Gelatin	Aladdin	CAS: 9000-70-8
Methanol	Aladdin	CAS: 67-56-1
2-Methylimidazole	Aladdin	CAS: 693-98-1
Multi-carboxylated carbon nanotubes	Aladdin	CAS: 308068-56-6
Cobalt (II) nitrate hexahydrate	Aladdin	CAS: 10026-22-9
Polyvinyl alcohol	Aladdin	CAS: 9002-89-5

**Software and algorithms**		

Origin 2021	OriginLab Corporation	https://www.originlab.com
MDI Jade 6.5	Materials Data Corporation	https://www.materialsdata.com/prodjd.html
Avantage	Thermo Fisher Scientific	https://mymicroscope.thermofisher.com

### Method details

#### Experiments

##### Materials

Gelatin (Gel, pharmaceutical grade, with a gel strength of approximately 240 g Bloom), methanol (CH_3_OH, superior grade pure, anhydrous, ≥99.9%, molecular weight: 32.04), 2-methylimidazole (C_4_H_6_N_2_, reagent grade, 98%, molecular weight: 82.10),Multi-carboxylated carbon nanotubes (CNTs, reagent grade, molecular weight: 12), cobalt (II) nitrate hexahydrate (Co(NO_3_)_2_·6H_2_O, reagent pure, 99.9%, molecular weight: 291.03), Polyvinyl alcohol (PVA, (C_2_H_4_O)_n_, analytical grade).

**Preparation of CNTs@ZIF-67 Composites:** “Initially, cobalt (II) nitrate hexahydrate and CNTs with different masses were dispersed in methanol to form mixed solutions with mass ratios (cobalt nitrate:CNTs) of 1:1, 1:1.5, and 1:2. The mixed solutions were then slowly added dropwise into a solution containing 2-methylimidazole under continuous stirring at room temperature for 24 h. Finally, the CNTs@ZIF-67 composites were obtained through centrifugation, washing, and drying of the samples.

**Fabrication of CNTs@ZIF-67 Electromagnetic Shielding Hydrogel:** First, the CNTs@ZIF-67 composites were uniformly dispersed in a solution containing gelatin (Gel) and polyvinyl alcohol (PVA) under ultrasonication. After thorough mixing, the homogeneous suspension was poured into molds and subjected to cyclic freeze-thaw treatment.

For the sake of convenience in writing, we name the PVA/Gel hydrogel as PG; the components added with ZIF-67 and CNTs are named PGZ and PGC, respectively; the hydrogels formed by three groups of CNTs@ZIF-67 composites prepared with different contents of CNTs are named PGZC1, PGZC2, and PGZC3, respectively.

**Tests and characterizations:** The hydrogel’s microstructural features were examined using Scanning Electron Microscopy (SEM). Fourier transform infrared spectroscopy was performed using an ATR-accessorized Nicolet iS 50 system. The number of scanning times was 32.X-ray diffraction (XRD) was carried out using a copper target at a measurement angle ranging from 5° to 90° and a scanning rate of 5°/min. Analyze the elements of the sample using X-ray Photoelectron Spectroscopy (XPS).

Differential scanning calorimetry (DSC) was employed to evaluate the thermal behavior of the hydrogel. The temperature change rate was 10 °C/min, which was applied for cooling crystallization and heating melting. The cylindrical - shaped hydrogel samples were prepared and subjected to compression tests using an electronic universal testing machine (in accordance with the execution standard: GB/T 1041–2008) to evaluate their compression performance. For the hydration level and swelling behavior tests of the hydrogel, the water content (θ) can be determined via straightforward measurement and application of the Equation 1^56^:

(Equation 1) $$\theta =\frac{Wa-We}{Wa}*100$$

Where Wa is the weight (g) of the sample in its original condition, and We refers to the weight (g) of the sample after it has undergone drying in an oven at 60°C for a period of 6 h to obtain the final dried sample. Meanwhile, the dried samples were used for the swelling property test. The dried samples were immersed in deionized water, and their weights were measured at different time intervals.

The electrical conductivity of a 3-millimeter-thick sample was measured using an ordinary four-probe resistance tester (model: FT-331, manufacturer: Ningbo Ruike Micro Intelligent Technology Co., Ltd.). The EMI shielding effectiveness (EMI SE)was estimated by a vector network analyzer (N5225B, keysight, Malaysia) within the X-band (8.2–12.4 GHz). The S11 and S21 (scattering parameters)of samples with a rectangular dimension were directly obtained, and then the power coefficients of reflectivity(R), transmissivity(T), and absorptivity(A), refection EMI SE(SE_R_), total EMI SE(SE_T_), and absorption EMI SE(SE_A_)were calculated using Equation 2, 3, 4, 5, and 6,^57^:

(Equation 2) $$R={\left|S_{11}\right|}^{2}$$

(Equation 3) $$T={\left|S_{21}\right|}^{2}$$

(Equation 4) A=1−T−R

(Equation 5) $${SE}_{R}=10\;\log\frac{1}{T}$$

(Equation 6) $${SE}_{A}=10\;\log\frac{1-R}{T}$$

### Quantification and statistical analysis

The figures in the main text were generated using Origin 2024 based on the raw data. X-ray photoelectron spectroscopy analysis was performed using Avantage.
