# Supplementary material for: CNTs@ZIF-67 conductive composite nanoparticles enhance the electromagnetic shielding performance of tough organic hydrogels
Source: iScience. 2026 Jan 7;29(11):114635. doi: 10.1016/j.isci.2026.114635 (PMC13676149; doi:10.1016/j.isci.2026.114635)
Supplement: Supplementary file 1 [file mmc1.pdf]

iScience, Volume *29*

Supplemental information

CNTs@ZIF-67 conductive composite nanoparticles enhance the electromagnetic shielding performance of tough organic hydrogels

Zhi Lei, Daohai Zhang, Yupeng Hu, Teng Zhou, Kunlan Diao, Dongju Liu, Anmin Song, Kaixiang Zou, and Shuhao Qin

Supplementary materials

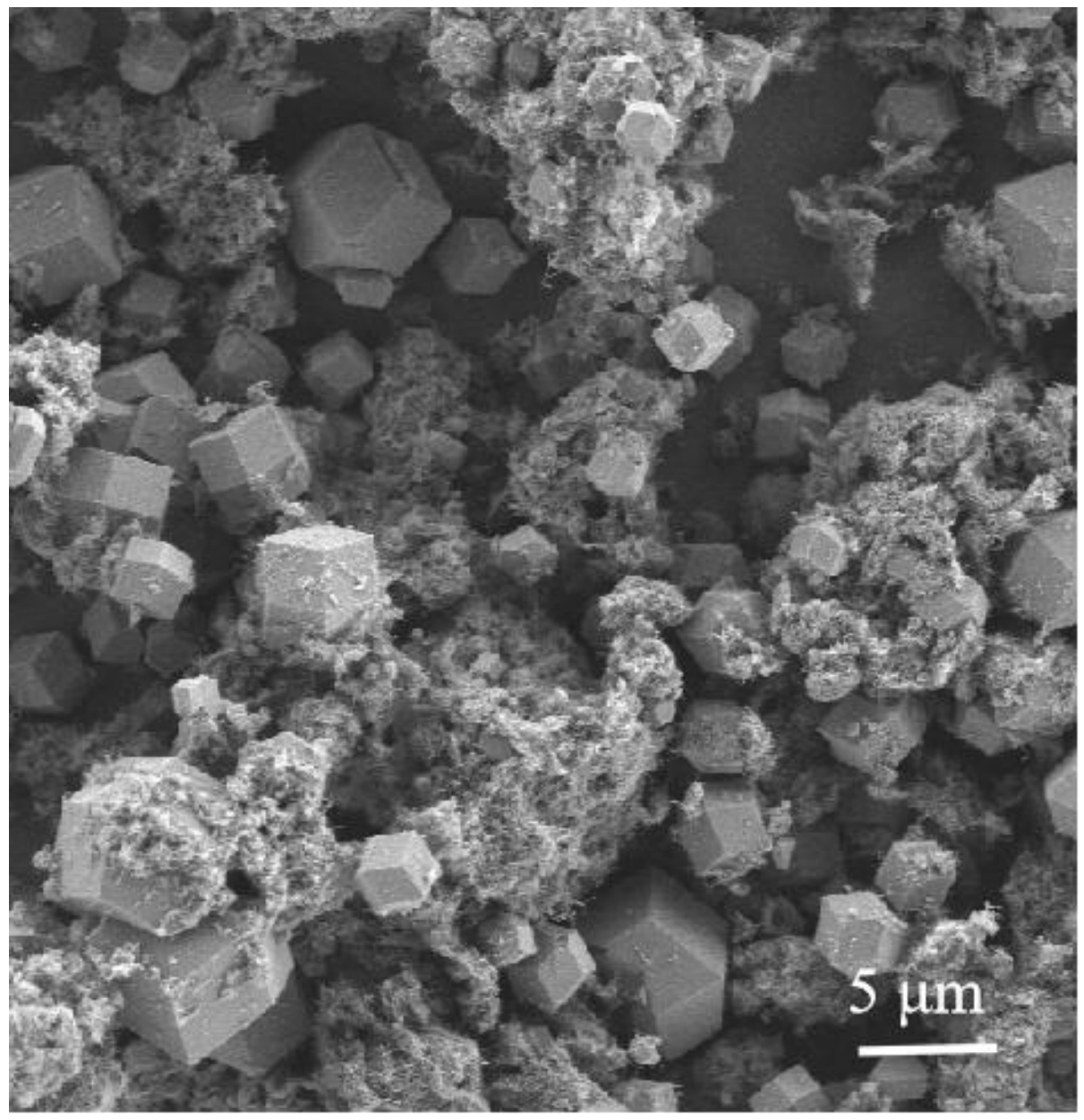

Figure S1. SEM image showing the surface morphology of CNTs@ZIF-67. (Scale bar: 5 μm.)

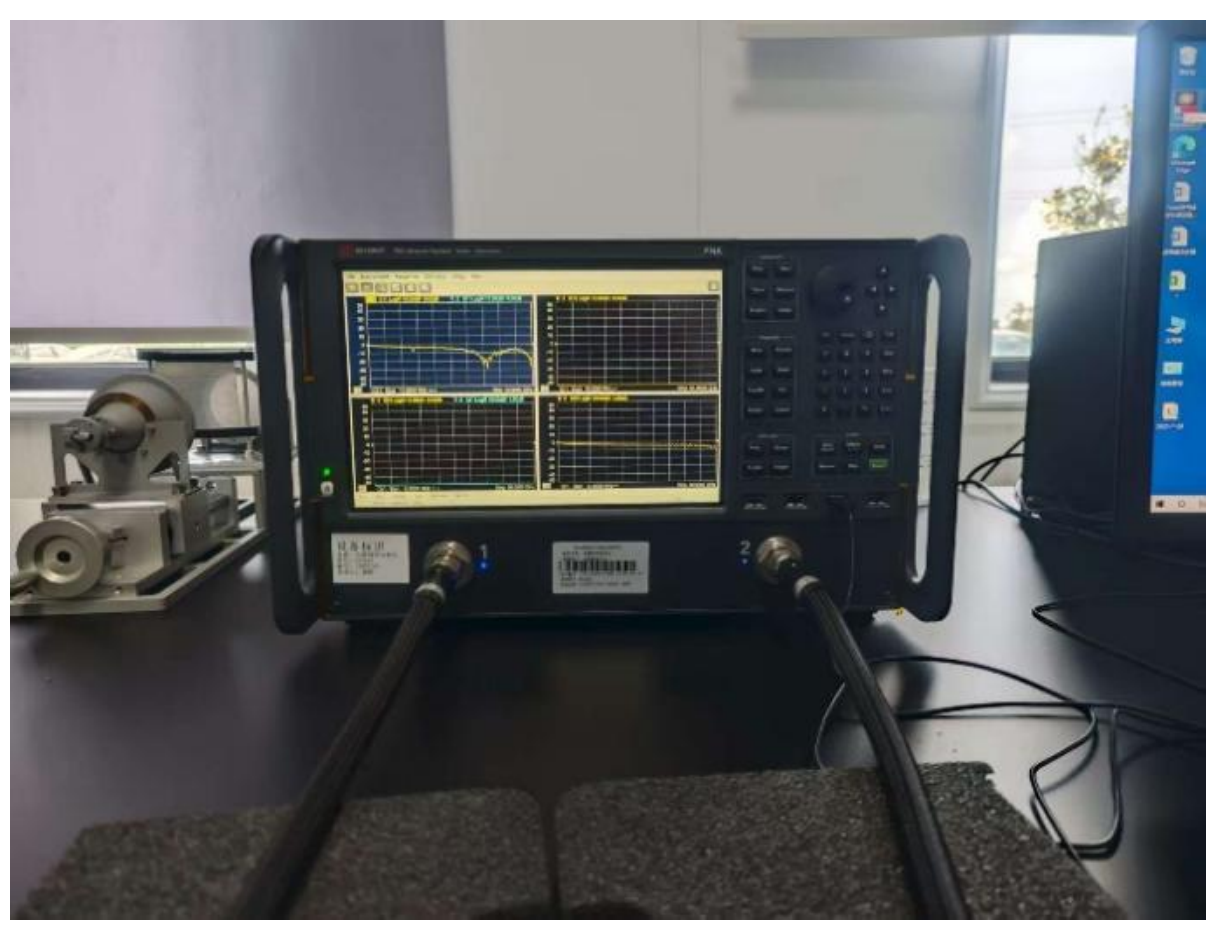

Figure S2. EMI shielding test equipment

Vector network analyzer (model: N5225B, manufacturer: Keysight, Malaysia)

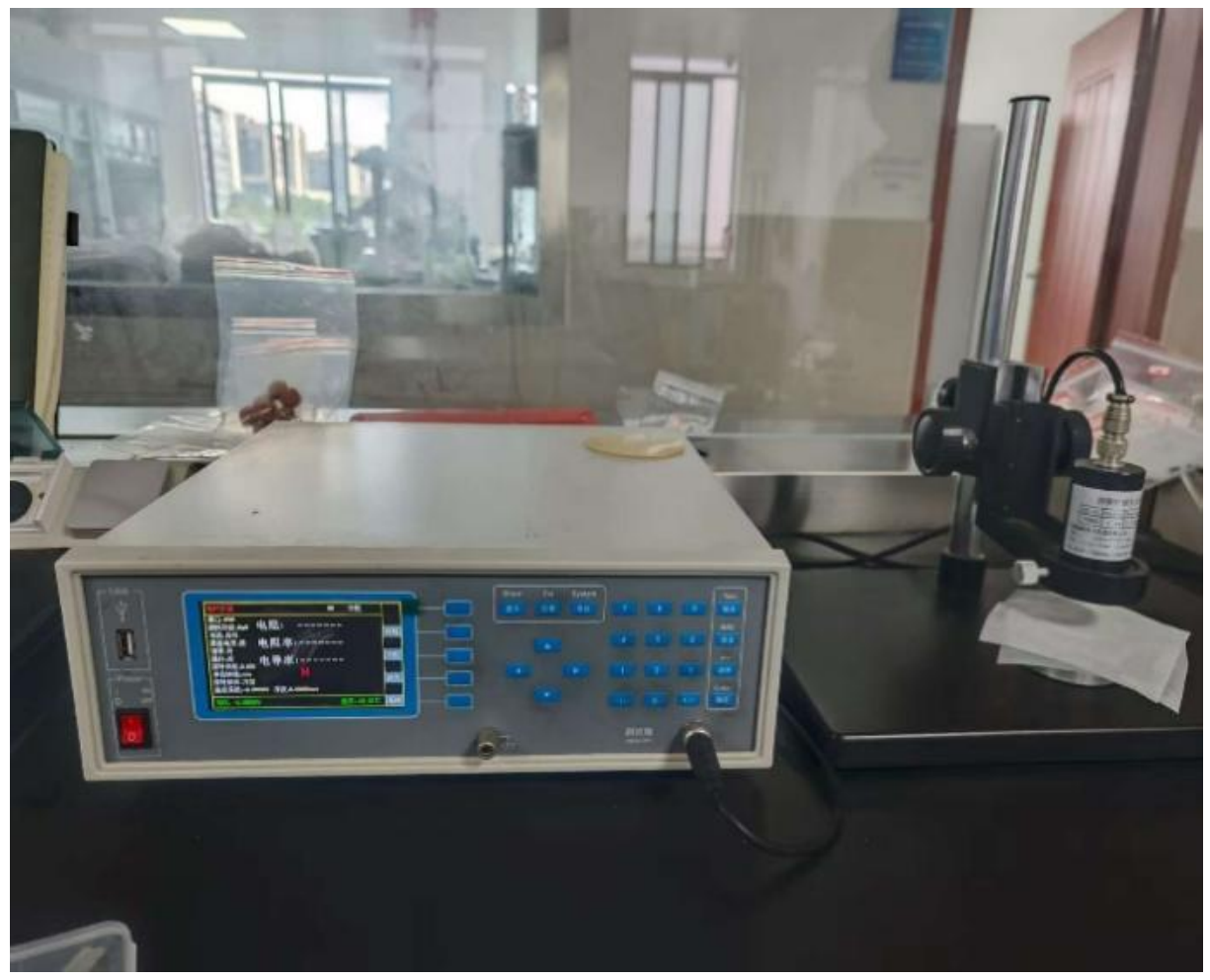

Figure S3. Four-point probe conductivity meter

Ordinary four-probe resistance tester (model: FT-331, manufacturer: Ningbo Ruike Micro Intelligent Technology Co., Ltd.) ； Four-probe probe (RadLus:0.5 mm, Spacing:2.35 mm, manufacturer: Ningbo Ruike Micro Intelligent Technology Co., Ltd.)
